# Reductionism redux

**DOI:** 10.7554/eLife.16964

**Published:** 2016-05-13

**Authors:** Christopher Miller

**Affiliations:** 1 Department of Biochemistry, Howard Hughes Medical Institute, Brandeis University, Waltham, United Statescmiller@brandeis.edu

**Keywords:** G protein gated potassium channel, G protein, ion channel, planar lipid bilayer, cardiac physiology, dopaminergic neuron, Human, Mouse

## Abstract

Experiments on artificial membranes are revealing many details about the workings of a family of potassium ion channels called GIRK channels.

**Related research articles** Touhara KK, Wang W, MacKinnon R. 2016. The GIRK1 subunit potentiates G protein activation of cardiac GIRK1/4 hetero-tetramers. *eLife* **5**:e15750. doi: 10.7554/eLife.15750

Wang W, Touhara KK, Weir K, Bean BP, MacKinnon R. 2016. Cooperative regulation by G proteins and Na^+^ of neuronal GIRK2 K^+^ channels. *eLife* **5**:e15751. doi: 10.7554/eLife.15751

**Image** GIRK channels are found in the heart and throughout the central nervous system

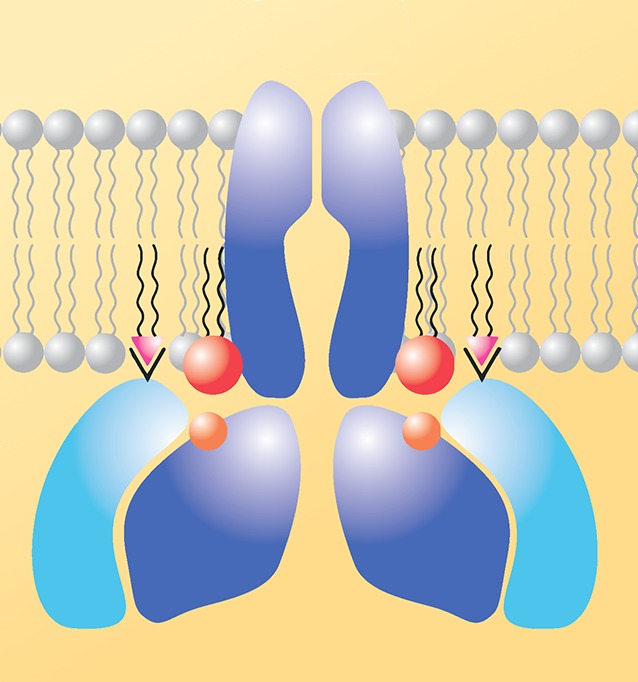

It's over a century since Henry Dale proposed that acetylcholine was a neurotransmitter (Dale, 1914), 95 years since Otto Loewi showed that the vagus nerve secretes acetylcholine to regulate the heartbeat (Loewi, 1921), 30 years since David Clapham and co-workers showed that acetylcholine binds to G-protein receptors, releasing the Gβγ subunits that activate the GIRK channels in cardiac cells (Logothetis et al., 1987), and three years since Rod MacKinnon and co-workers served up a crystal structure of a GIRK channel complexed with Gβγ subunits (Whorton et al., 2013). Over this span of time, Gβγ regulation of ion channel activity has spawned a field densely populated with neurobiologists and electrophysiologists, with GIRKs being a major focus because of their physiological importance, phenomenological richness, experimental tractability and linkage to human diseases.

Like all potassium ion channels, GIRK (short for G protein gated inward rectifier potassium) channels help shape cellular electrical signals by "quieting" the excitatory actions of sodium, calcium and other ion channels. Most GIRK channels contain four sub-units: those found in cardiac pacemaker cells contain either four GIRK4 sub-units, or two GIRK1 sub-units and two GIRK4 sub-units, and channels containing four GIRK2 sub-units are found in neurons. The Gβγ subunit is also molecularly diverse, with at least 5 β and 13 γ isoforms known. What strikes me as truly remarkable for such intensely studied channels is that the energetics of GIRK activation by Gβγ has remained almost entirely unknown.

Now, in two papers in eLife, MacKinnon and co-workers offer a close-up thermodynamic view of how Gβγ 'ligands' switch on their GIRK 'receptors' in two mammalian systems: the GIRK1/4 channels found in cardiac pacemaker cells and the GIRK2 channels found in neurons. The experiments on GIRK1/4 were performed by Kouki Touhara, Weiwei Wang and MacKinnon, at Rockefeller University (Touhara et al., 2016), while those on GIRK2 were performed by Wang, Touhara and MacKinnon in collaboration with Keiko Weir and Bruce Bean of Harvard Medical School (Wang et al., 2016).

By analyzing a web of binding and conformational equilibria, the researchers produce detailed, yet clear and intuitive, answers to a series of fundamental questions about the activation of GIRK channels. How tightly does the activator bind? How many of the four Gβγ sites in the channel must be occupied for opening to occur? What sort of crosstalk operates between Gβγ and other GIRK activators, such as the phosphoinositides that reside in the cell membrane or the sodium ions (Na^+^) found in the cytoplasm? How much Gβγ is released into the cell membrane in a synaptic response? Why are certain GIRKs modulated by Na^+^ and others not? Such questions had not been answered previously, and those few that were posed at all produced soft or discordant conclusions. These questions have lingered so long for a simple reason: the complexity of cell membranes.

The currents through GIRK channels are governed by a sequence of largely uncontrolled time-dependent processes, even in experiments involving an isolated membrane patch facing a solution containing purified Gβγ. The process that causes the most difficulty is probably the hydrolysis of PIP2 (a lipid that is found in cell membranes) by lipid phosphatases embedded in the membrane. And it is no help that soluble Gβγ, though experimentally convenient, only qualitatively mimics the diffusion of this ligand in two dimensions in the cellular membrane.

To explore the properties of ion channels researchers often work with "reconstituted" systems in which purified ion channels are inserted into artificial membranes (see, for example, Wang et al., 2014). MacKinnon and co-workers used membranes that contained known amounts of PIP2 and employed an elegant trick to systematically vary the "concentration" of Gβγ in these membranes (Figure 1). This allowed them to probe the activity of the GIRK channels as the concentration of Gβγ was varied.

**Figure 1.**
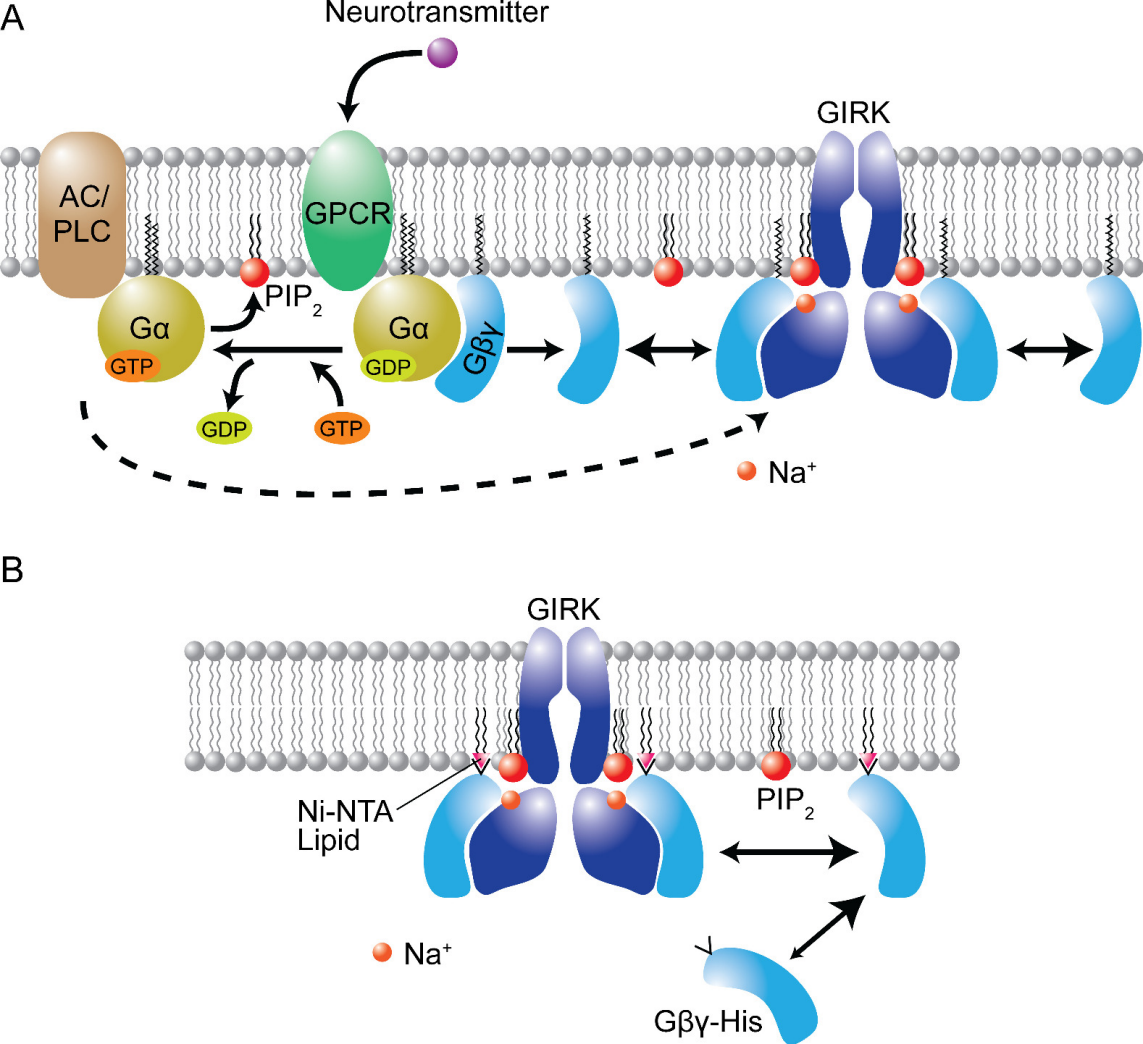
Cellular complexity reduced in a reconstituted membrane. (**A**) Cellular signaling pathway for the activation of a GIRK channel. A neurotransmitter (such as acetylcholine) binds to its receptor (GPCR), thus releasing the membrane-associated Gα and Gβγ subunits of a G-protein. The Gα subunit activates adenylate cyclase (AC) and phospholipase C (PLC), modulating the level of PIP2 in the cell membrane; the Gβγ subunit activates the GIRK channel. (**B**) The reduced systems studied by MacKinnon and co-workers are composed of lipid bilayers that contain a GIRK channel and ~1% of PIP2 lipid. The surface density of Gβγ subunits depends on the amount of Ni-NTA lipid in the bilayer because the Gβγ subunits (which are soluble) have a tag (His) that makes them bind to the Ni-NTA lipids. Image courtesy of Weiwei Wang.

The two papers establish a parade of fundamental insights into GIRK channel operation. First, the channels open only when all four sites are occupied by Gβγ, which binds with "positive cooperativity" such that the affinity of the fourth ligand is higher than the affinity of the first by a factor of ~40. These features are functional manifestations of what we know about the structure of the GIRK2–Gβγ complex from X-ray diffraction studies (the Gβγ-binding surface becomes more exposed as the channel opens; Whorton et al., 2013), and they ensure that the channel opens in a switch-like manner as the cellular stimulus is increased. Second, although it is specific, Gβγ binding is also quite weak, so the channels are poised to close swiftly after removal of the stimulus. Third, Na^+^ in the physiological range boosts Gβγ affinity to GIRK2 or GIRK4 channels: this allows the GIRK channels to increase their activity when floods of Na^+^ ions enter neurons during high-frequency bursts of excitation.

Touhara et al. then went on to explore why GIRK1/4 channels are not sensitive to cytoplasmic Na^+^, whereas GIRK4 channels are. The Na^+^-binding site in the GIRK4 subunit contains an aspartic acid (Asp) residue, but there is an asparagine (Asn) residue at the same site in the GIRK1 subunit (Ho and Murrell-Lagnado, 1999). Na^+^ binding increases Gβγ affinity in GIRK4 channels, and therefore increases responsiveness to stimulation of the G protein. Touhara et al. find that GIRK1/4 channels behave as if permanently in a high-Na^+^ state, even when the concentration of Na^+^ is zero, as though the Asn residue of GIRK1 mimics the electrically neutral Asp-Na^+^ configuration of the GIRK4 channel at high levels of Na^+^. Touhara et al. confirm that GIRK currents in cardiac cell lines are Na^+^-insensitive, implying that most of the GIRK4 in these cells is tied up with GIRK1 in GIRK1/4 channels. Finally, this new understanding of Na^+^ modulation reported in the first paper (Touhara et al., 2016) was cleverly exploited in the second paper (Wang et al., 2016) to estimate the absolute surface density of Gβγ in dopamine neurons at the peak of a physiological GIRK response: about 1 subunit per 10,000 Å^2^.

These papers offer a crisp illustration of the power of using purified, defined components to reconstitute membrane functions. Although reductionist approaches are sometimes dismissed as 'unphysiological', they can answer questions about which complex cellular systems are stubbornly ambiguous. The picture of GIRK activation emerging from these experiments can now be fed back into the cellular context with much more confidence than has ever before been possible.
